# Neutrophils in Cancer: Two Sides of the Same Coin

**DOI:** 10.1155/2015/983698

**Published:** 2015-12-24

**Authors:** Eileen Uribe-Querol, Carlos Rosales

**Affiliations:** ^1^División de Estudios de Posgrado e Investigación, Facultad de Odontología, Universidad Nacional Autónoma de México, 04510 Ciudad de México, Mexico; ^2^Departamento de Inmunología, Instituto de Investigaciones Biomédicas, Universidad Nacional Autónoma de México, 04510 Ciudad de México, Mexico

## Abstract

Neutrophils are the most abundant leukocytes in blood and are considered to be the first line of defense during inflammation and infections. In addition, neutrophils are also found infiltrating many types of tumors. Tumor-associated neutrophils (TANs) have relevant roles in malignant disease. Indeed neutrophils may be potent antitumor effector cells. However, increasing clinical evidence shows TANs correlate with poor prognosis. The tumor microenvironment controls neutrophil recruitment and in turn TANs help tumor progression. Hence, TANs can be beneficial or detrimental to the host. It is the purpose of this review to highlight these two sides of the neutrophil coin in cancer and to describe recent studies that provide some light on the mechanisms for neutrophil recruitment to the tumor, for neutrophils supporting tumor progression, and for neutrophil activation to enhance their antitumor functions.

## 1. Introduction

Neutrophils are the most abundant leukocytes in blood and are considered to be the first line of defense during inflammation and infections [1]. Invading microorganisms evoke an inflammatory response that recruits neutrophils from the circulation into the tissues. There, neutrophils destroy the microorganism by a series of mechanisms, mainly phagocytosis, release of antimicrobial substances, and the formation of neutrophil extracellular traps (NETs) [2]. Activated neutrophils also release proteinases into the surrounding tissue, causing damage to the host [3]. In addition, neutrophils are capable of producing many cytokines and chemokines, which can influence the inflammatory response, as well as the immune response [4, 5].

Besides this classical role in antimicrobial functions, neutrophils are also found infiltrating many types of tumors. Early studies suggested that these tumor-associated neutrophils (TANs) were mere bystanders because it was hard to imagine that neutrophils, being short-lived cells, could have an effect on chronic and progressive diseases such as cancer. However, more recently it is becoming clear that TANs have relevant roles in malignant disease. This renewed interest comes in part from the recognition that cancer-related inflammation is an important feature for the development of many tumors [6] and it is a hallmark of cancer [7]. Indeed, neutrophils may be potent antitumor effector cells [8]. The various antimicrobial and cytotoxic compounds contained in granules can destroy malignant cells, and cytokines and chemokines secreted by neutrophils can also recruit other cells with antitumor activity [5, 9].

However, an increasing number of clinical observations and laboratory studies have shown that presence of neutrophils in tumors correlates with poor prognosis. This has been well documented for bronchoalveolar carcinoma [10], melanoma [11], renal carcinoma [12], and head and neck squamous cell carcinoma (HNSCC) [13]. In all these cases, neutrophils display a protumor phenotype that could be adverse to the host. The tumor microenvironment controls neutrophil recruitment and in turn TANs help tumor progression. TANs are different from circulating neutrophils (as discussed later), and, in untreated tumors of murine models, they can display a protumorigenic phenotype. The mechanisms for this phenotype are just beginning to be elucidated, but some of them involve genotoxicity, angiogenesis, and immunosuppression [8]. Hence, tumor-associated neutrophils can be beneficial or detrimental to the host [14]. These two types of TANs described in mice have been named N1 and N2 [15] in a similar manner as antitumor and protumor macrophages (TAMs) [16].

It is the purpose of this review to highlight these two sides of the neutrophil coin in cancer and to describe recent studies that provide some light on the mechanisms for neutrophil recruitment to the tumor, for neutrophils support to the tumor, and for neutrophil activation to enhance their antitumor functions and in the future improve cancer immunotherapy.

## 2. Neutrophils in Cancer

Our knowledge on the role of neutrophils in human cancers is relatively small. From an initial interest in the 1980s, the number of publications on neutrophils in cancer-related studies has been steadily going down [14]. However, this trend is now beginning to change with the realization that neutrophils are indeed important players in cancer development, as reflected by several recent reviews [16–18], and as we will see next.

In many patients with advanced cancer, elevated counts of neutrophils in blood are found. How tumors induce neutrophilia is uncertain, but production of granulocyte-macrophage colony-stimulating factor (GM-CSF) is a possible mechanism in several types of cancer [19]. In addition, other cytokines such as granulocyte colony-stimulating factor (G-CSF), interleukin- (IL-) 1, and IL-6 produced by tumors seem to contribute to elevated neutrophil numbers in blood [20]. This neutrophilia is associated with poor prognosis in several types of cancers, such as lung, melanoma, and renal carcinomas [11, 21, 22]. In agreement with this, the presence of neutrophils within certain tumors seems also to be an indicator of poor prognosis. Reduced recurrence-free time and overall survival were reported for neutrophil-infiltrated tumors in renal carcinomas [12], HNSCC [13], pancreatic adenocarcinomas [23], and liver carcinoma [24]. Because neutrophilia is frequently associated with inflammatory responses to infections and tissue damage, neutrophilia represents evidence for the concept of cancer-related inflammation inducing tumor progression [7].

### 2.1. The Neutrophil-to-Lymphocyte Ratio (NLR)

The relation of neutrophil numbers in blood to other leukocyte counts has been suggested to serve as a prognostic factor for cancer. Thus, the neutrophil-to-lymphocyte ratio (NLR) was introduced as prognostic factor for colorectal cancer [25]. Due to its simplicity, NLR has shown to be a readily available and inexpensive biomarker for many types of tumors including non-small-cell lung cancer [26], hepatocellular carcinoma [24], nasopharyngeal carcinoma [27], colorectal cancer [28], melanoma [11], and breast cancer [29, 30]. In general, the blood NLR is elevated in patients with more advanced or aggressive disease, as indicated by increased tumor size, nodal stage, and number of metastatic lesions [31]. Also, a high NLR correlates with adverse overall survival in many solid tumors [32, 33].

Despite the clinical evidence from the many studies mentioned above, neutrophilia (larger numbers of neutrophils in blood as a consequence of elevated egress of cells from the bone marrow) is not always a bad indicator for cancer progression. In some types of tumors, for example, gastric cancer, an elevated neutrophil blood count is indicative of positive prognosis [34]. This means that neutrophils can control cancer in some instances. In fact, the capacity of neutrophils to directly kill tumor cells both* in vitro* and* in vivo* was reported long time ago [35–37]. Also, neutrophils from tumor-bearing animals were reported to have enhanced cytotoxic activity [38, 39]. And recently, neutrophils isolated from blood of some healthy individuals presented direct cytotoxicity against several tumor cell lines [40]. Therefore, the exact role of neutrophils within the tumor is a controversial matter [14, 41].

### 2.2. Myeloid-Derived Suppressor Cells (MDSCs)

In addition to the elevated number of neutrophils in blood, an increase in the frequency of immature myeloid cells at earlier stages of differentiation has also been detected in several types of tumors [42], including terminal patients with lung, breast, and gastrointestinal cancer [43]. These immature cells consist of a heterogeneous population of immunosuppressive cells defined as myeloid-derived suppressor cells (MDSCs) [44]. These MDSCs can be divided phenotypically into granulocytic (G-MDSC) and monocytic (Mo-MDSC) subgroups [45, 46] and are found in great numbers in the spleens of tumor-bearing animals, where they display an immunosuppressive phenotype helping tumor progression [47, 48]. The G-MDSCs have immature neutrophil morphology and the consensus phenotype CD33^+^/CD11b^+^/HLA-DR^lo/−^/CD15^+^ in humans [49]. They have been found in peripheral blood of patients with glioblastoma [50], multiple myeloma [49], Hodgkin lymphoma [51], or head and neck cancer [52].

These MDSCs present various mechanisms of immunosuppression. The main mechanism involves production of reactive oxygen species (ROS) by the respiratory burst of these cells. In advanced cancer patients, the hydrogen peroxide (H_2_O_2_) produced by activated granulocytes reduced expression of the T cell receptor (TCR) CD3 *ζ* chain and decreased cytokine production by patients' T cells [53]. These oxidized human T cells had defective chemotaxis and presented impaired F-actin remodeling. The effect was found to be mediated by oxidation of the actin-remodeling protein cofilin [54]. Cofilin is activated through dephosphorylation at Ser3, and then it mediates severing and depolymerization of F-actin for formation of the immune synapse and T cell activation. Cofilin oxidation induced formation of an intramolecular disulfide bridge that prevents its activation, thus leading to impaired T cell activation [54]. Also, long-term oxidative stress leads to translocation of cofilin into the mitochondria and necrotic-like programmed cell death takes place in human T cells [55]. In addition, exposure of ROS to memory/effector CD45RO^+^ T cells results in inhibition of NF-*κ*B activation and reduction in Th1 cytokines production [56]. Furthermore, MDSC-produced ROS can lead to CD8^+^ T cell tolerance by another mechanism involving peroxynitrite [57]. ROS can combine with nitric oxide and form peroxynitrite, which is highly reactive at short distances. During MDSC-T cell contact, peroxynitrite induces nitration of the T cell receptor and CD8 molecules. This process makes CD8-expressing T cells unable to bind peptide-MHC complexes and to respond to the specific peptide [57].

Another mechanism for T cell suppression is production of Arginase 1 (ARG1) by MDSCs. ARG1 inhibits T cell proliferation by degrading extracellular arginine, which results in decreased responsiveness of T cells to CD3/TCR stimulation [58]. For example, in patients with non-small cell lung cancer, TANs had reduced intracellular ARG1 and tumor-infiltrating lymphocytes showed reduced proliferation in response to CD3/TCR stimulation. All non-small cell lung cancer cell lines secreted IL-8, and IL-8 was effective in triggering ARG1 release [59]. Also, in patients with glioblastoma, degranulated neutrophils associated with elevated levels of serum ARG1 correlated with decreased T cell CD3 zeta chain expression in peripheral blood T cells, resulting in immunosuppression [60]. Together, these mechanisms explain how MDSC-produced ROS and ARG1 mediate T cell suppression in cancer settings.

Because, G-MDSCs share many properties with neutrophils [61] but seem to be functionally different from mature neutrophils [13, 62], a transcriptomic analysis was conducted to compare in tumor-bearing animals circulating neutrophils with TANs and with MDSCs [63]. It was concluded that indeed TANs are not “tissue-based” G-MDSC but a distinct population of neutrophils [63]. However, at present it is not clear whether TANs are mature neutrophils or represent a special category of cells such as immature neutrophils with protumor properties.

### 2.3. Phenotypes of Tumor-Associated Neutrophils (TANs)

Depending on the phenotype displayed by TANs, they have been classified in tumor-bearing mice as N1 or N2 [15]. Similarly to antitumor tumor-infiltrating macrophages (M1), N1 cells display proinflammatory and antitumorigenic functions. In contrast, M2 and N2 cells display protumorigenic activity [16]. TANs seem to be different from circulating neutrophils and also from G-MDSC in the bone marrow and spleen [44, 63]. Upon transforming growth factor-beta (TGF-*β*) blockade, murine CD11b^+^/Ly6G^+^ neutrophils recruited to tumors were hypersegmented and more cytotoxic to tumor cells and expressed higher levels of proinflammatory cytokines [15]. In contrast, depletion of these neutrophils decreased tumor growth and resulted in more activated CD8^+^ T cells intratumorally. Thus, it seems that TGF-*β* within the tumor microenvironment induces a population of TANs with a protumor phenotype [15]. In support of this idea, in two models of murine tumor cancer cell lines (Lewis lung carcinoma and AB12 mesothelioma), neutrophils were found primarily at the periphery of the tumor at early stages of tumor development. These TANs were more cytotoxic toward tumor cells and produced higher levels of tumor necrosis factor-alpha (TNF-*α*), NO, and H_2_O_2_. In contrast, TANs in established tumors had these functions downregulated and presented a more protumorigenic phenotype [64]. These results showed that neutrophils enter the tumor and become more protumor with tumor progression [64]. Therefore, murine TANs can have an antitumorigenic (N1) phenotype but also a protumorigenic (N2) phenotype capable of supporting tumor growth and suppressing the antitumor immune responses [14, 41], depending on the tumor microenvironment [17].

Despite this classification, the nature and function of TANs in the cancer microenvironment remain largely unknown, particularly with human tumors. However, two recent publications describe the phenotype of neutrophils infiltrated into human tumors. In one study of surgically resected lung cancer patients, TANs were isolated from digested human lung tumors and constituted 5%–25% of the cells in the tumor. These TANs presented an activated phenotype (CD62L^lo^/CD54^hi^) with expression of a distinct repertoire of chemokine receptors that included CCR5, CCR7, CXCR3, and CXCR4 [65]. In addition, TANs produced larger quantities of the proinflammatory factors MCP-1, IL-8, MIP-1*α*, and IL-6 than blood neutrophils did. TANs could also stimulate T cell proliferation and interferon gamma (IFN-*γ*) release. These results indicate that, in the earliest stages of lung cancer, TANs are not immunosuppressive but rather stimulate T cell responses [65]. In the second study, the role of chronic inflammation, particularly via IL-23 and IL-17, in developing human colorectal cancer was investigated. Authors found that innate *γδ*T (*γδ*T17) cells were the major cellular source of IL-17 in colorectal cancer. Tumor growth led to epithelial barrier disruption allowing microbial products to induce inflammatory dendritic cell accumulation and *γδ*T17 polarization in human tumors. These activated dendritic cells induced *γδ*T17 cells to secrete IL-8, TNF-*α*, and GM-CSF, thus leading to accumulation of neutrophils in the tumor. These TANs were characterized by CD45^+^/Lin^−^/HLADR^−^/CD11b^+^/CD33^+^/CD66b^+^ and displayed typical polymorphonuclear morphology. Thus, they were described as G-MDSC [66]. These TANs (G-MDSC) produced much more ARG1 and ROS than autologous neutrophils and inhibited proliferation of activated autologous T cells and IFN-*γ* production [66].

The TANs described in these reports show that in human tumors the dual role of neutrophils is also observed. In early tumors, TANs seem to be able to stimulate T cell responses [65], but later in established tumors TANs are immunosuppressive [66]. These important reports are just “the tip of the iceberg” in our understanding of the origin and function of TANs. Many questions remain—for example, are TANs in early tumors mature neutrophils with antitumor properties and TANs in established tumors immature cells (G-MDSCs) with immunosuppressive properties directly recruited from the circulation? Or are TANs mature neutrophils that develop a more protumor phenotype with tumor progression?—as suggested by several tumor animal models and cancer patients [17, 64]. A very recent publication identifies several subpopulations of neutrophils in the blood of tumor-bearing mice and in human cancer patients and describes several relationships of these cells in connection to cancer progression [67].

In this study, subpopulations of circulating neutrophils in cancer animals could be distinguished according to their densities. One subpopulation is composed of “normal” high-density neutrophils (HDNs). The other subpopulation has lower density neutrophils (LDNs) that copurify with the low-density mononuclear cells layer formed when separating leukocytes by density gradient centrifugation [68]. In tumor-free mice, most neutrophils were HDNs, but in tumor-bearing animals LDNs increased progressively and often became the dominant neutrophil type in circulation [67]. The HDNs from cancer animals, which were previously reported as tumor-entrained neutrophils (TENs) [69], displayed high cytotoxicity toward tumor cells in culture, whereas LDNs were not cytotoxic [67]. Also, the LDNs had reduced expression of various chemokines (CXCL1, CXCL2, CXCL10, CCL2, and CCL3) and chemokine receptors (CXCR2 and CCR5), consistent with a reduced inflammatory state. The LDN subpopulation consists of large mature (fully formed lobulated nucleus) neutrophils and also of immature neutrophils, similar to G-MDSC. The authors then showed by BrdU labeling that LDNs rapidly accumulate in the circulation, whereas HDNs appear in the circulation much later. This is consistent with the idea that some of the LDNs are indeed immature neutrophils. In addition, authors showed that HDNs are capable of becoming LDNs upon treatment with TGF-*β* [67]. It is interesting to note that TGF-*β* was able to induce the change of HDNs from tumor-bearing mice into LDNs, but it had no effect on HDNs from tumor-free mice [67]. This indicates that other stimuli are also needed for this change in animals with cancer. For example, treatment of naïve healthy mice with recombinant G-CSF protein elicited G-MDSC similar to those induced in tumor-bearing animals [70]. Together, all these results support a model proposed by the authors, in which neutrophils are present in three subpopulations in cancer: normal high-density neutrophils, immature low-density neutrophils (G-MDSC), and large mature low-density neutrophils. These cell types present diversity in function and plasticity. While the HDNs are antitumor and the LDNs are protumor [67], they can change under the influence of the different chemokines and cytokines in the tumor microenvironment [17].

## 3. Recruitment

**Figure 1:**
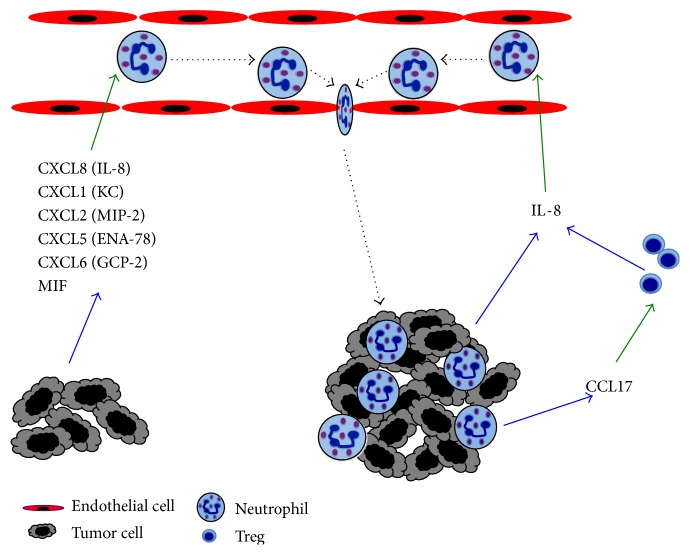
Mechanisms of neutrophil recruitment to tumors. Tumor cells produce many chemokines, such as CXCL1 (KC), CXCL2 (MIP-2), CXCL5 (ENA-78), CXCL6 (GCP-2), CXCL8 (IL-8), and MIF, which are chemoattractants for neutrophils. These cells then migrate out of the blood circulation into the tumor. Tumor-associated neutrophils can also produce CCL17, an important chemoattractant for regulatory T cells (Treg). These inhibitory Treg in turn produce more IL-8, the most potent chemoattractant for neutrophils, creating a positive loop for more neutrophil infiltration into the growing tumor. Blue arrows denote molecules secreted by cells. Green arrows denote the action of molecules on cells. Dotted lines denote cell movement.

Solid tumors are composed of several cell types, including tumor cells and stromal cells. The tumor stroma contains fibroblasts, endothelial cells of blood vessels, and in many cases immune cells. These tumor-infiltrating immune cells highlight the inflammatory microenvironment that is commonly associated with tumor progression [7]. In addition to lymphocytes and macrophages, neutrophils are found in great numbers in a wide variety of tumors [12, 13, 23, 24]. Clearly, neutrophils are recruited to the tumor by the action of neutrophil-attracting chemokines that can be produced not only by other immune cells but also directly by several tumor cells (Figure 1). The most effective neutrophil chemokine is interleukin-8 (IL-8/CXCL8). It was found that oncogenic Ras induced IL-8 expression [71], and Ras-expressing mouse adenomas produced KC/CXCL1 and MIP-2/CXCL2, the murine equivalents of IL-8, to attract TANs [72]. These findings suggested that TANs are recruited to help the tumor. Accordingly, increased IL-8 levels were found in HNSCC patients [13], and elimination of neutrophils in cancer murine models reduced tumor burden [73] and metastasis [74]. Deleting IL-8 receptors also reduced tumor growth [75]. These findings support the notion that tumor-produced IL-8 is important for neutrophil recruitment to help tumor progression [76]. However, the IL-8 receptors CXCR1 and CXCR2 are also expressed on other cell types including endothelial cells and tumor cells. Thus, determining the extent of neutrophil involvement in IL-8-mediated tumor progression will require future studies.

Using the same CXCR1 and CXCR2 receptors, neutrophils can also respond to other chemokines such as CXCL1, CXCL2, CXCL5, CXCL6, and CXCL7 [77] (Figure 1). CXCL2 can induce neutrophil infiltration in tumors, and it was suggested that this is an autocrine effect [78]. Supporting this idea, it was also found that in TANs the expression of CXCL2, and also CXCL1, was upregulated more than 150-fold [63]. Therefore, it seems that neutrophils activate a positive feedback mechanism by releasing neutrophil chemokines that attract more neutrophils into the tumor, similarly to neutrophil recruitment into sites of infection [79]. The role of ENA-78/CXCL5 in appearance of TANs in carcinoma of the liver was investigated in 919 patients with hepatocellular carcinoma. CXCL5 was found to be overexpressed in patients with recurrent tumors, and the levels of CXCL5 correlated with greater appearance of TANs and with shorter overall survival [80]. Another chemokine that also participates in neutrophil recruitment to tumors is GCP-2/CXCL6. In a melanoma mouse model, specific anti-CXCL6 monoclonal antibodies reduced the number of TANs and also tumor size [81]. In addition, migration inhibitory factor (MIF), another tumor-derived chemokine for neutrophils, was identified in HNSCC tumors. MIF was described as an inhibitor of macrophage migration* in vitro*, but it is now known that it also binds CXCR2 [82] (Figure 1). Tumor-derived MIF levels correlated with higher TANs levels and poor survival of these patients [83].

Many tumor cells can directly produce chemokines for neutrophils, but various other cells within the tumor may also be the source for these chemokines and other cytokines. In particular, activated T cells are known to produce GM-CSF, CXCL1, CXCL2, TNF-*α*, and IFN-*γ* [84]. These factors could directly or indirectly recruit more neutrophils to the tumor. Although the influence of activated T cells in neutrophil recruitment to tumors is not known, regulatory T cells (Treg) seem to be important for neutrophil infiltrating tumors. In one study, Treg were found to inhibit neutrophil recruitment to a tumor site by reducing the expression of CXCL1 and CXCL2 [85]. In contrast, in another study, Treg promoted neutrophil infiltration to tumors by producing IL-8 [86]. Thus, the influence of T cell function on the appearance of TANs needs to be further explored. In addition, TANs can also recruit more Treg. Murine TANs secrete CCL17, a potent chemokine for Treg, at higher levels than circulating or splenic neutrophils [87] (Figure 1). Moreover, the amounts of CCL17 increased progressively during tumor progression. It seems then that TANs and Treg act together to impair antitumor immunity [87].

## 4. Protumor Function of Neutrophils

A large body of clinical evidence indicates that neutrophils are involved in cancer development and tumor progression. In most cases, large numbers of TANs are associated with advanced disease and poor prognosis for cancer patients. This negative association has been reported for several solid tumors, such as melanoma, hepatocellular carcinoma, non-small cell lung carcinoma, glioma, HNSCC, adenocarcinoma, and colon cancer [41, 88].

**Figure 2:**
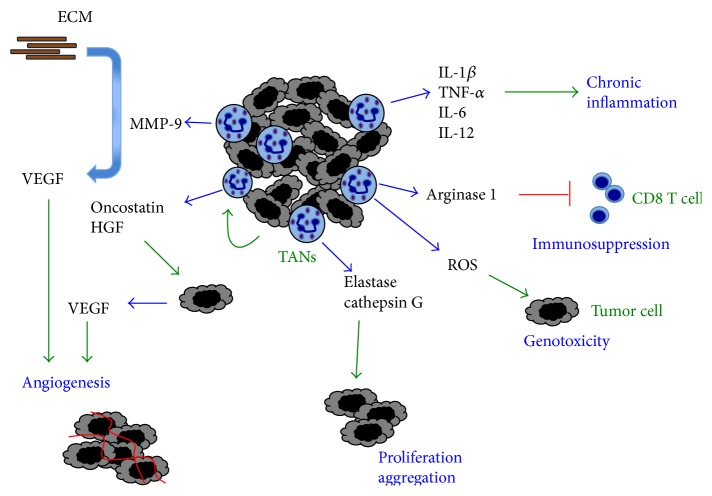
Protumor activity of neutrophils. Tumor-associated neutrophils (TANs) help tumor progression in several ways. TANs can secrete matrix metalloproteinase-9 (MMP-9) that releases vascular endothelial growth factor (VEGF) from the extracellular matrix (ECM) to promote angiogenesis. TAN can secrete cytokines (IL-1*β*, TNF-*α*, IL-6, and IL-12) that induce a chronic inflammatory state and arginase 1, which inhibits CD8 T cells, creating an immunosuppressive state. TANs also produce reactive oxygen species (ROS) that can damage DNA, inducing genotoxic effects on tumor cells. Serine proteases, such as elastase and cathepsin G, from neutrophil granules seem to have a direct effect on tumor cells for inducing proliferation. Certain tumors, like breast cancer cells, induce neutrophils to produce Oncostatin, an IL-6-like cytokine that then stimulates breast cancer cells to secrete vascular endothelial growth factor to promote angiogenesis (red lines represent new blood vessels). Also, hepatocellular carcinoma cells induce neutrophils to release hepatocyte growth factor (HGF), which activates tumor cells to become more invasive. Blue arrows denote molecules secreted by cells. Green arrows denote the action of molecules on cells.

Neutrophils display several protumor functions. Most of them have just recently begun to be revealed. These functions involve the same molecules neutrophils use to destroy microorganisms and to modulate inflammation. Important molecules that can modify growth and invasiveness of tumors involve granule proteins, matrix-degrading proteinases, reactive oxygen species (ROS), chemokines, and cytokines. Recent reports describe how TANs use these molecules to affect cell proliferation, angiogenesis, metastasis, and immune surveillance (Figure 2).

### 4.1. Neutrophil Molecules

#### 4.1.1. Neutrophil Elastase

Neutrophil elastase (NE) is a major protein of azurophilic granules that is released upon cell degranulation. The main physiologic function of NE seems to be elimination of invading microorganisms [89], but it also has important inflammatory effects [3]. NE is a serine protease with a broad range of substrates; among them are neutrophil-derived antibacterial proteins, extracellular matrix proteins, integrins, cytokines, and cytokine receptors. In addition to its roles in inflammation and bacteria destruction, NE has presented various protumor effects both* in vivo* and* in vitro* [90]. NE was found to directly promote A459 tumor cell proliferation when murine neutrophils were cocultured with this lung carcinoma cell line [91]. This effect was markedly reduced when tumor cells were cocultured with NE^−/−^ neutrophils, or in the presence of an NE inhibitor. The effect of NE on tumor growth was dependent on phosphatidylinositol 3-kinase (PI-3K), since it was also reduced in the presence of a PI-3K inhibitor [91]. Staining experiments showed that NE got inside the tumor cells via clathrin-coated pits and localized at early endosomes [92]. Once inside the cell, NE acted on insulin receptor substrate-1 (IRS-1). Because IRS-1 binds to the regulatory unit of PI-3K, its degradation by NE led to more PI-3K available to enhance the proliferation pathway [93]. Similar results have been found with other types of tumor cells, including esophageal cancer [94], gastric cancer [95], and breast cancer [96]. In these cases, NE mediated release of transforming growth factor-alpha (TGF-*α*) from the cell surface. Furthermore, NE has also been found to promote migration of tumor cells. Coculture of human neutrophils with pancreatic ductal adenocarcinoma cells (PDAC) resulted in dyshesion of cells from the monolayer. The same effect was observed by adding NE to PDAC cultures and correlated with loss of surface expression of E-cadherin [97]. NE also enhanced the migratory capacity of esophageal cancer cells [94].

#### 4.1.2. Cathepsin G

Cathepsin G is a peptidase from azurophilic granules that participates in degradation of phagocytosed microorganisms and in remodeling of extracellular matrix (ECM) proteins [98]. Also, cathepsin G can promote angiogenesis and tumor cell migration [99–101]. Breast cancer MCF-7 cells form spherical cell aggregates when incubated with neutrophils. This process involves cell adhesion via E-cadherin and requires cathepsin G [99]. Moreover, the process has been shown to involve two steps. First cathepsin G binds to the tumor cell surface, independently of its catalytic site, and then induces cell aggregation, which is dependent on its enzymatic activity [99] (Figure 2). Cathepsin G degrades ECM molecules such as fibronectin and attenuates binding between integrins and fibronectin. This leads to E-cadherin-mediated homotypic cell-cell adhesion, which is protease-resistant [101]. The formation of these tumor cell aggregates would allow tumor cells to disseminate via the circulation to distant sites and establish new metastases. Once at the new site, tumor cells would need new vasculature. In a model of breast cancer metastasis to the bone, it was also found that cathepsin G enhanced TGF-*β* signaling and upregulated vascular endothelial growth factor (VEGF) to promote angiogenesis [100]. Together, these reports indicate that TANs-derived cathepsin G may induce ECM remodeling and promote tumor progression and metastasis [102, 103].

#### 4.1.3. Matrix Metalloproteinase-9

Matrix metalloproteinase-9 (MMP-9/gelatinase B) is released from secondary (specific) granules and is believed to help neutrophils in the process of extravasation via degradation of ECM proteins. MMP-9 was found to promote tumor proliferation in a human papilloma virus- (HPV-) 16 skin carcinogenesis model. MMP-9^−/−^ mice showed reduced keratinocyte proliferation, but this phenotype was reversed when bone marrow-derived leukocytes were transplanted into irradiated mice [104]. Also, immunostaining of MMP-9 in squamous cell carcinoma tumors showed that MMP-9 was found only in tumor infiltrating leukocytes and not in tumor cells [104]. In addition, MMP-9 has been shown to inhibit apoptosis of tumor cells in the lung [105]. Thus, MMP-9 supplied by bone marrow-derived cells is responsible for enhancing tumor proliferation via both increased proliferation and reduced apoptosis of tumor cells.

Another important effect of MMP-9 that supports tumor growth is angiogenesis. The vascular endothelial growth factor (VEGF) is sequestered in the ECM after it is produced by cells (Figure 2). The proteolytic release of VEGF from tissue ECM via MMPs is regarded as a prerequisite for* in vivo* induced angiogenesis [106, 107]. The angiogenic effect of MMP-9 has been reported in several cancer models. Melanoma cells were transfected to overexpress the GCP-2/CXCL6 chemokine and then implanted into nude mice. The new CXCL6-melanoma tumors grew larger and with a well-developed vasculature than wild type (WT) melanomas [108]. These larger tumors also presented higher levels of MMP-9 and induced a strong influx of TANs [108]. Similarly, in a model of pancreatic adenocarcinoma, new dysplastic lesions that develop into carcinomas are formed with enhanced angiogenesis. This process has been named the angiogenic switch [109]. In these new lesions, MMP-2 and MMP-9 were upregulated. MMP inhibitors and genetic ablation of MMP-9 reduced the angiogenic switching, tumor number, and tumor growth [109], indicating that MMP-9 can render normal islets angiogenic. In addition, malignant keratinocyte transplantation resulted in tumors with neutrophils expressing predominantly MMP-9 and stromal cells expressing mainly MMP-2 and MMP-3 [110]. Depletion of a singular MMP did not affect neovascularization of malignant murine keratinocytes.

These reports suggested a direct role for MMP-9 in tumor angiogenesis, but they did not identify the cell type producing this protease. Reconstitution of tumor-bearing MMP-9^−/−^ mice with wild type, MMP-9-competent hematopoietic cells demonstrated that tumor-infiltrating myeloid cells were the source for MMP-9 [111, 112]. In a murine model of pancreatic islet carcinogenesis, MMP-9-expressing neutrophils were predominantly found inside angiogenic islet dysplasias and tumors, whereas MMP-9-expressing macrophages were localized along the periphery of such lesions. Transient depletion of neutrophils significantly reduced the frequency of initial angiogenic switching in dysplasias [113]. Also TANs in melanoma or fibrosarcoma tumors expressed high levels of MMP-9 and VEGF, and elimination of these TANs resulted in reduced tumor growth [114]. Also, reducing TANs in prostate carcinoma tumors reduced angiogenesis and tumor cell intravasation [115]. Moreover, in cancer patients, neutrophils expressing high levels of MMP-9 have also been found. In HNSCC, expression of MMP-9 was larger by TANs than by any other cell type in the tumor [116], and in hepatocellular carcinoma larger numbers of TANs correlated with more angiogenesis [117, 118]. Direct proof for neutrophils being the major tumor-associated leukocyte type expressing MMP-9 was recently provided in a study employing human xenografts and syngeneic murine tumors [119]. When tumors or isolated TAMs and TANs were double-stained for MMP-9 and for respective macrophage- or neutrophil-specific antigens, only TANs gave a strong signal for MMP-9 [119, 120]. In addition, it was calculated that 1 × 10^6^ neutrophils or TANs could release approximately 100–200 ng proMMP-9 within 1-2 h of incubation. In contrast, 1 × 10^6^ macrophages or TAMs would require several weeks to produce the same amount of proMMP-9 [119, 120]. Hence, neutrophil-derived MMP-9 is responsible for enhancing angiogenesis via release of VEGF from the ECM in many types of tumors (Figure 2).

The unusual angiogenic potency of neutrophil MMP-9 is related to its unique way of production. In other cell types, the zymogen proMMP-9 is released together with the inhibitor of metalloprotease 1 (TIMP-1), which slows the activation of MMP-9 and can also inhibit the proteolytic activity of the once activated enzyme [121]. Therefore, the TIMP-1-free proMMP-9 from neutrophils can be activated easier and function much longer than MMP-9 from other cell types [122, 123].

#### 4.1.4. Reactive Oxygen Species

Neutrophils are efficient producers of reactive oxygen species (ROS) for destruction of microorganisms. ROS can also indirectly promote tumor growth. First, neutrophils generate hydrogen peroxide (H_2_O_2_), which is next converted to hypochlorous acid (HOCl) by myeloperoxidase (MPO). HOCl can then activate several ECM-degrading MMPs, including MMP-2, MMP-7, MMP-8, and MMP-9. Also, HOCl can block TIMP-1 and in this manner potentiate the proteolytic activity of MMPs [124, 125]. Finally, as indicated above, MMP activity leads to enhanced tumor progression by inducing proliferation and angiogenesis.

Nevertheless, a more potent and direct effect of ROS on tumor cells is genotoxicity, which might lead to carcinogenesis (Figure 2). Although neutrophil-derived ROS and HOCl can directly damage and destroy tumor cells, they can also cause genotoxicity in circumstances when they do not kill cells. ROS-mediated genotoxicity is induced by two major pathways: oxidative DNA damage and MPO catalyzed activation of chemical carcinogens [126]. Point mutations and DNA strand breaks are induced in many different cell types when cocultured with neutrophils [126], and HOCl has been reported to be mutagenic in lung epithelial A549 cells [127].

#### 4.1.5. Arginase 1 (ARG1)

Upon release from neutrophil granules, ARG1 gets activated to degrade extracellular arginine, an essential amino acid for proper activation of T cells. Thus, degranulation of neutrophils may exert an immunosuppressive effect in tumors by inhibiting T cells in a similar manner to the one described for G-MDSC [88]. In fact, depletion of TANs in tumor-bearing animals increased the numbers of activated CD8^+^ T cells and promoted smaller tumors [15]. Similarly, non-small cell lung cancer cells stimulated neutrophils through IL-8 to release ARG1, and in tumors TANs had reduced levels of ARG1 [59]. More recently, the same group found that ARG1 released from gelatinase granules was inactive at physiological pH unless activated by factor(s) stored in azurophil granules [58]. Thus, TANs can induce ARG1-dependent immunosuppression through concomitant exocytosis of gelatinase and azurophil granules (Figure 2).

#### 4.1.6. Cytokines

Neutrophils can also produce cytokines or growth factors, which increase the tumorigenic potential of cancer cells [5]. Two clear examples have been described for Oncostatin-M [128–130] and for hepatocyte growth factor [10, 131, 132]. Breast cancer cells can stimulate neutrophils to release Oncostatin-M, an IL-6-like cytokine. Oncostatin-M in turn stimulated breast cancer cells to secrete VEGF [133] (Figure 2). Similarly, hepatocellular carcinoma cells stimulated neutrophils to release hepatocyte growth factor (HGF). In turn, HGF stimulated tumor cells to become more invasive [134] (Figure 2).

### 4.2. Metastasis

Neutrophils can also influence the migration potential of cancer cells. In several types of cancer it has been shown that neutrophils promote metastasis. These tumors include skin squamous cell carcinoma [135], melanoma [136], adenocarcinomas [137], HNSCC [83], and breast cancer [138]. The way neutrophils augment the migratory capacity of tumor cells involves many different mechanisms that are just beginning to be elucidated.

Tumors can induce activation of neutrophils to release inflammatory factors that promote tumor migration. In HNSCC, tumor-derived MIF not only recruits TANs but also induced these cells to display promigratory effects on the tumor cells [83]. Similar responses have been documented for different cancer cell lines but through a different mediator. Various tumor cells release hyaluronan, which can then activate neutrophils via TLR4 and the PI-3K/Akt signaling pathway. In turn, neutrophils induce enhanced migration of the tumor cells [139].

**Figure 3:**
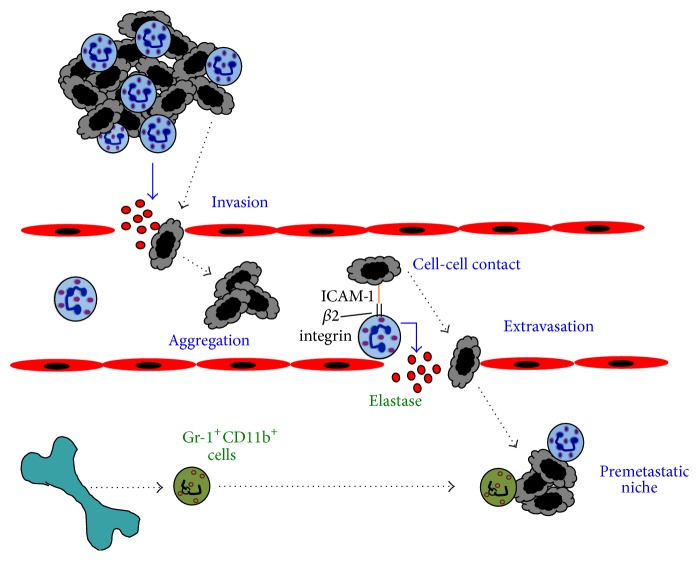
Neutrophils can promote tumor cell invasion and metastasis. Tumor-associated neutrophils (TANs) help tumor invasion in several ways. TANs can secrete enzymes, such as elastase (red dots), that degrade the basement membrane and promote tumor cell invasion through the basement membrane. Once in circulation, neutrophils can also help tumor cells to survive by inducing tumor cell aggregation. Circulating tumor cells can directly adhere to arrested neutrophils via the adhesion molecule ICAM-1 on the tumor cells, and *β*2 integrins on neutrophils. This cell-cell interaction promotes extravasation of the tumor cells. Bone marrow-derived cells including neutrophil precursors (Gr-1^+^CD11b^+^ cells) migrate to premetastatic niches where they secrete factors that promote tumor cell growth. Blue arrows denote molecules secreted by cells. Dotted lines denote cell movement.

Very early reports suggested that TANs release enzymes that degrade the basement membrane and promote tumor cell invasion through the basement membrane [137] (Figure 3).* In vitro* studies showed that human neutrophils assist the human breast tumor cell line MDA-MB-231 to cross a monolayer of endothelial cells [140]. Tumor cell-conditioned medium downregulated neutrophil cytotoxicity and upregulated expression of adhesion molecules, facilitating tumor cell migration. In contrast, MDA-MB-231 cells alone did not transmigrate [140]. Also, in the presence of neutrophils, melanoma cell adhesion and transmigration through an endothelial cell monolayer were increased [141, 142] (Figure 3). This process seems to involve at least in part the protease NE, which can induce severe tissue damage, and as mentioned before correlates with poor prognosis [90]. Elevated amounts of NE in various types of cancer can induce tumor invasion and metastasis by degrading ECM proteins [143]. In support of this, it was reported that inhibition of NE could reduce metastasis to the liver [144].

Once in circulation, neutrophils can also help tumor cells to survive by inducing tumor cell aggregation (Figure 3). In patients with breast and prostate cancers, tumor cell clusters in blood have been associated with poor survival [145], and in animal models, injection of tumor cell clusters resulted in more metastases than injection of dispersed tumor cells [146]. At least, for breast cancer MCF-7 cells, neutrophils can promote aggregation* in vitro* [99, 101]. However, metastasis induced by neutrophil-mediated aggregation of tumor cells has not yet been directly demonstrated* in vivo*.

Circulating tumor cells directly adhere to the vascular endothelium promoting extravasation for establishing new metastases. At the site of exit, lung cancer tumor cells have been observed in close association with neutrophils [147]. In this process, neutrophils enhance tumor cell retention and in consequence induce more metastasis [148] (Figure 3). Direct cell-cell interaction of neutrophils with breast carcinoma cells has been shown to involve the adhesion molecule ICAM-1 on the tumor cells and *β*2 integrins on neutrophils. Neutrophils bound tumor cells engaging integrins and inducing ICAM-1 clustering on the tumor cell [148] (Figure 3). This activated in the tumor cell a signaling pathway involving focal adhesion kinase (FAK) and p38-MAPK that resulted in enhanced migration [138]. In addition, this enhanced migration was shown* in vivo* to result in increased metastasis to the liver [149]. Here, the cancer cells adhered directly on top of arrested neutrophils, which acted as a bridge to facilitate interactions between the tumor cells and the liver parenchyma [149].

Moreover, neutrophils seem to participate in facilitating metastasis even before the tumor cells arrive to the new site, the metastatic niche. This is a potential metastatic site where leukocytes create a permissive growth environment prior to the arrival of tumor cells [150, 151]. VEGFR1-positive bone marrow-derived cells are found in premetastatic niches of organs involved in metastasis of particular tumor types [152]. Once at the metastatic niche, these bone marrow-derived cells secrete factors that promote tumor cell growth [152, 153] (Figure 3). In lungs of mice bearing mammary adenocarcinomas, Gr-1^+^CD11b^+^ cells were significantly increased before tumor cells arrived. These granulocytic cells had decreased IFN-*γ* production and increased MMP-9 production, thus promoting angiogenesis [154]. In addition, coinjection of with 4T1 tumor cells with these Gr-1^+^CD11b^+^ cells, isolated from tumors and spleens of 4T1 mammary tumor-bearing mice, resulted in increased metastases to lungs [155]. But because these Gr-1^+^CD11b^+^ cells are a heterogeneous population of cells, including neutrophils, macrophages, dendritic cells, and other immature myeloid cells, the particular cell type(s) needed to promote metastasis remains unclear. However, neutrophils are a good candidate because it has been reported that circulating neutrophils augment in number with increasing metastatic potential of various rat mammary adenocarcinomas [156], and tumors secreting IL-8 also have an increased metastatic potential [124]. Clearly, the mechanisms TANs use to promote tumor cell migration and metastasis are diverse and complex (Figure 3).

## 5. Antitumor Function of Neutrophils

Despite the large amount of evidence for a negative role of neutrophils during tumor progression, there is also clear evidence for a positive role of neutrophils in carcinogenesis. As mentioned before, neutrophils can display antitumor activity in different forms. Early murine neutrophils infiltrating tumors have been named N1 since they clearly display an active proinflammatory and an antitumor phenotype [15]. In fact, the antitumor capacity of neutrophils has been recognized for more than three decades. Neutrophils can directly kill tumor cells both* in vitro* [36] and* in vivo* [37].

Neutrophils potentiate this antitumor effect when they have been activated. For example, a colon adenocarcinoma cell line transfected to express G-CSF lost tumorigenic activity after considerable concentration of neutrophils at the tumor site [157]. Interestingly, neutrophils could discriminate between G-CSF-producing and G-CSF-nonproducing cells and directly inhibited only G-CSF-producing tumor cells [157]. This antitumor effect of activated neutrophils can also be transferred to other animals, as demonstrated with spontaneous regression/complete resistance (SR/CR) mice. SR/CR mice resist very high doses of cancer cells that are lethal to WT mice even at low doses. The genetic, cellular, and molecular effector mechanisms in this model are largely unknown. However, purified neutrophils from the SR/CR mice independently killed cancer cells* in vitro* and completely transferred resistance to WT recipient mice [158]. Also, the cancer disappeared gradually following infiltration of a large number of neutrophils and few lymphocytes into the remaining tumor tissues [159]. The importance of N1 type TANs in antitumor responses is also highlighted by reports showing that depletion of murine neutrophils results in enhanced tumor growth [15, 160, 161].

Despite the evidence presented before on neutrophils helping metastasis by preparing the metastatic niche, a complete opposite effect has also been demonstrated for metastatic breast cancer [69] and renal carcinoma [162]. In both models, neutrophils prevented metastasis to the lung. In the breast cancer model, the tumor cells produced CCL2 that induced neutrophil ROS production [69], while, in the renal carcinoma model, tumor-derived IL-8 recruited tumor cytotoxic neutrophils [162]. This goes against the majority of reports implicating IL-8 in protumor functions of neutrophils. Nevertheless, these findings underline the dual antitumor and protumor potential of neutrophils and suggest that neutrophils could be induced to enhance their antitumor responses.

### 5.1. Mechanisms of Tumor Killing

**Figure 4:**
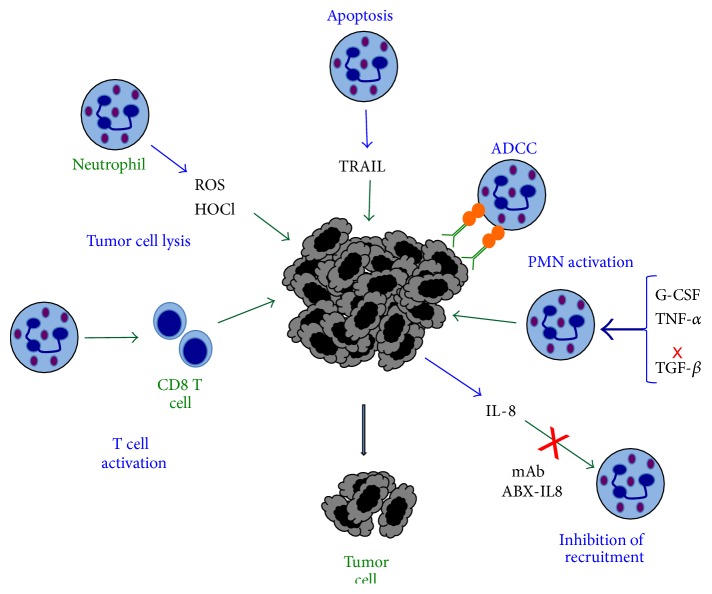
Antitumor activity of neutrophils. Neutrophils produce reactive oxygen species (ROS) and hypochlorous acid (HOCl) that can directly damage and destroy tumor cells. By direct contact or by release of TRAIL, neutrophils can also induce apoptosis of certain tumor cells. The most effective antitumor mechanism is antibody-dependent cell-mediated cytotoxicity (ADCC). Antibody molecules (green) that bind to tumor antigens are recognized by Fc receptors (orange circles) on neutrophils. This binding activates a cytotoxic response against the tumor cell. Neutrophils can be activated to display a stronger antitumor phenotype with granulocyte colony-stimulating factor (G-CSF), transforming growth factor-*α* (TNF-*α*), or by blocking (red cross) transforming growth factor-*β* (TGF-*β*). Also, the blockage of IL-8, with specific monoclonal antibodies (such as mAb ABX-IL8), can prevent new neutrophil infiltration into growing tumors. Inflammatory neutrophils can also activate cytotoxic (CD8) T cells. All these mechanisms result in smaller tumors. Blue arrows denote molecules secreted by cells. Green arrows denote the action of molecules on cells.

Neutrophils clearly have the potential of directly killing tumor cells. The mechanisms by which neutrophils accomplish this function are numerous and not completely understood, but they involve many of the same antimicrobial and immune regulatory functions of neutrophils (Figure 4).

#### 5.1.1. ROS

Early reports indicated that neutrophils from tumor-bearing animals displayed enhanced superoxide anion generation and phagocytosis. This led to reduced tumors and less metastatic foci in lungs [38, 39]. Also, it has been shown that indeed ROS produced by neutrophils can induce tumor cell lysis, through HOCl delivered directly at the cell membrane [163]. Although ROS could be genotoxic for tumor cells, it is clear that, in the case of rapidly growing tumors, activated neutrophils producing sufficient singlet oxygen can eliminate tumor cells at the early phase of tumor development [164] (Figure 4).

#### 5.1.2. Direct Lysis and Apoptosis

Because neutrophils require close contact mediated by integrins to induce killing, it is also possible that neutrophils may induce direct lysis of tumor cells by a mechanism similar to the one used by NK cells via the enzymes perforin and granzyme [165]. However expression of these enzymes in neutrophils is controversial [166, 167]. Neutrophils can also induce certain tumor cells to undergo apoptosis. Neutrophils induced apoptosis of human breast cancer cells, when stimulated by antibodies targeted to HER-2 [168].

#### 5.1.3. TRAIL

Neutrophils have another way of eliminating tumor cells by inducing apoptosis of the malignant cell. This effect is mediated by the tumor necrosis factor-related apoptosis inducing ligand (TRAIL) (Figure 4). For a long time, carcinoma* in situ* of the bladder has been treated with intravesical administration of* Mycobacterium bovis* bacillus Calmette-Guérin (BCG). This kind of immunotherapy is very effective for treatment of this type of cancer [169], but the mechanism is only partially known [170].

It was then found that neutrophils in urine of patients with carcinoma of the bladder and under BCG immunotherapy expressed high levels of TRAIL [171]. Neutrophils from these patients can selectively induce apoptosis of tumor cells [172]. TRAIL is expressed on these neutrophils at high levels both as a type II membrane protein (intracellular amino terminal portion and carboxyl terminus outside the cell) and as a biologically active soluble form [173], which is released from intracellular stores after interaction with components of the BCG cell wall [174].

TRAIL is a member of the TNF family of molecules, known to have apoptosis-inducing functions [175]. TRAIL binds to target cells through two death receptors (DRs) (DR4/TRAIL-R1 and DR5/TRAIL-R2) and three decoy receptors (DcRs) [DcR1/TRAIL-R3, DcR2/TRAIL-R4, and osteoprotegerin] [176]. DRs activate the formation of a death-inducing signaling complex for caspase activation and initiation of apoptosis [177].

An important feature of neutrophil TRAIL-induced apoptosis is that it can kill tumorigenic and transformed cells but not normal cells and tissues [170, 178]. For this reason, TRAIL is becoming a major physiologic weapon against cancer [172], and several research laboratories and pharmaceutical companies are developing recombinant forms of TRAIL or TRAIL receptor agonists for therapeutic purposes [178]. In addition, the importance of TRAIL in other clinical conditions, such as infectious diseases, autoimmunity, and cardiovascular diseases, is becoming more apparent. Therefore, understanding the regulatory mechanisms of TRAIL signaling will help in the future to control these health problems [178].

#### 5.1.4. Matrix Metalloproteinase-8

Neutrophils can protect against some tumors by secreting MMP-8. In mice deficient in MMP-8, an increase in skin tumors with an increase in neutrophil infiltrates to the tumors was reported [179]. This protective effect is not clearly defined, but it involves the inhibition of neutrophil migration into the tumor site.

#### 5.1.5. Antibody-Dependent Cell-Mediated Cytotoxicity

Antibodies directed to tumor cells can also bind to Fc receptors on the membrane of immune cells [180]. In many cases, the antibody activates these cells to destroy the tumor cell. This antibody-dependent cell-mediated cytotoxicity (ADCC) is capable of eliminating efficiently various types of tumors. NK cells are particularly efficient in this response via the Fc*γ* receptors [181] (Figure 4). Neutrophils also present efficient ADCC against cells that have been marked by antibodies [182]. However, the mechanism of killing is not completely described, but it seems to be different from the classic ADCC mechanism used by NK cells.

It is worth noting that an important difference exists between murine and human neutrophils regarding Fc*γ*R expression [183]. In addition to Fc*γ*RIII, the only Fc*γ* receptor on murine NK cells, murine neutrophils also express Fc*γ*RIV. In contrast, human neutrophils express two unique Fc*γ* receptors not present in other species: Fc*γ*RIIa (CD32a) (homolog to murine Fc*γ*RIII) and Fc*γ*RIIIb (CD16b) (a glycosylphosphatidylinositol- (GPI-) linked receptor). Human NK cells only express Fc*γ*RIIIa (homolog to murine Fc*γ*RIV) [183]. Therefore special attention should be paid when interpreting data from murine models on ADCC against tumors. Human neutrophils present a more efficient ADCC when they engage Fc*γ*RIIa [184, 185]. Under stimulated conditions mainly with IFN-*γ* but also with G-CSF, neutrophils can upregulate expression of Fc*γ*RI (CD64). This receptor seems also capable of promoting neutrophil ADCC against tumors [186] and in particular with squamous head and neck cancer [187]. However, in other studies, it was shown that immature neutrophils with high expression of Fc*γ*RI had reduced ADCC activity via this receptor [188]. In fact, ample reports have demonstrated that the high affinity receptor for IgA, Fc*α*RI (CD89), is a more potent inducer of ADCC by neutrophils [188, 189].

The mechanism for tumor cytotoxicity from neutrophils is not completely known, and it seems to be multifactorial. Both ROS-dependent and ROS-independent mechanisms have been suggested for neutrophil ADCC [190]. For the oxidative mechanism, close cell contact mediated by integrins is required for direct release of HOCl to the tumor cell [163]. However, studies with neutrophils from chronic granulomatous disease (CGD) patients and with ROS scavengers suggest that ROS are not as important for ADCC as they are for antimicrobial functions [191]. Another proposed mechanism is direct cell lysis via perforin and granzyme [165]. However, as mentioned before, expression of these enzymes in neutrophils remains controversial [166, 167].

#### 5.1.6. Regulation of T Cell Function

Neutrophils invading tumors can modify T cell effector functions and in this way instruct T cells to reject tumors. Cytotoxic CD8^+^ T cells are key contributors in any immune response towards tumors. As mentioned before N2 neutrophils can be inhibitors of T cell functions [15, 192]. However, the proinflammatory N1 neutrophils can recruit and activate CD8^+^ T cells [15, 193] (Figure 4). Also, after photodynamic therapy, there was a rapid neutrophil infiltration into the treated tumor bed. Neutrophils were necessary for generation of tumor-specific primary and memory CD8^+^ T cell responses [160]. Together, these reports indicate that neutrophils can influence the outcome of T cell functions depending on the type of cytokines they produce [4, 194].

## 6. Neutrophil Extracellular Traps

Neutrophil extracellular traps (NETs) constitute a recently described form of the antimicrobial arsenal of neutrophils. NETs are fibers of chromatin released from neutrophils in an active process named NETosis [195]. In this process, neutrophils undergo dramatic changes starting with flattening of the cells. Next, chromatin decondensation with histone modifications takes place. Citrullination of histone H3 by peptidylarginine deiminase 4 (PAD4) is a major modification during NETosis. The nucleus loses its typical lobular morphology and the nuclear membrane disappears. Finally, DNA is released from the cell [196]. DNA fibers in NETs are also decorated with various antimicrobial proteins from the neutrophil granules, including neutrophil elastase, MPO, cathepsin G, proteinase 3, MMP-9, and bactericidal/permeability increasing protein (BPI) [197]. NETs form a mesh-like structure where microorganisms get trapped and are either directly killed on some cases or more often subsequently phagocytosed by other neutrophils [198, 199].

Many microorganisms and various stimuli can directly stimulate NET formation. Bacterial products such as lipopolysaccharide (LPS), formyl-methionyl-leucyl-phenylalanine (fMLF), and also phorbol esters such as phorbol myristate acetate (PMA) are efficient NET inducers [200]. Recent reports also indicated that antigen-antibody complexes are capable of inducing NET formation, thus suggesting a direct role for Fc receptors in this function [201]. In fact both Fc*α*RI [202] and Fc*γ*RIIIb [203] have been shown to induce NET formation [204]. Also, some cytokines such as TNF-*α* and IL-8 can also enhance NET formation [205]. This is interesting because various tumors are known to produce these cytokines and thus it is possible that tumors can enhance NET formation. However, this idea has not yet been proved in any type of cancer.

### 6.1. NETs in Tumors

The role of NETs in cancer is just beginning to be elucidated. Very little is known about the presence and effect of NETs in different types of tumors. It is also not clear if distinct TANs can make NETs with different efficiency. In an initial study, tumor samples from eight patients with Ewing sarcoma were evaluated for the presence of TANs and NETs, defined as extracellular staining for MOP. In two (25%) patients, intratumoral NETs were found. After surgery these patients presented early relapse. Thus, it was proposed that at least this type of tumor could induce TANs to release NETs [206]. This idea has not been confirmed in other types of cancer. However, in cancer models of chronic myelogenous leukemia and mammary and lung carcinoma, peripheral neutrophils were more prone to NET formation. Neutrophils from tumor-bearing animals responded to platelet-activating factor (PAF) forming more NETs than neutrophils from tumor-free animals. In addition, higher amounts of circulating neutrophils and plasma cell-free DNA were found in tumor-bearing animals [207]. This free DNA is probably in the form of NETs, since a concomitant increase in neutrophils with hypercitrullinated histone H3 was also found [207]. It seems then that some cancers may present a systemic effect on the host that predisposes neutrophils to form NETs.

**Figure 5:**
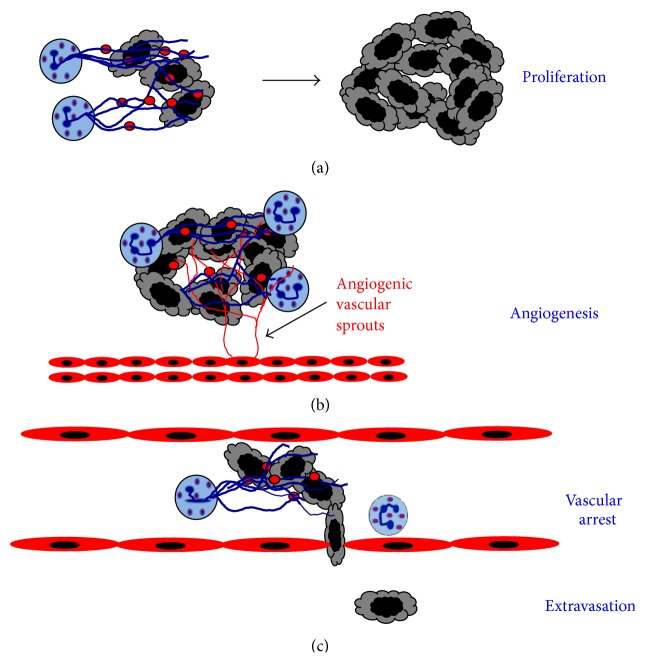
Neutrophil extracellular traps (NETs) can induce tumor progression. Tumor-associated neutrophils can produce NETs (blue lines), which are chromatin fibers decorated with proteins from neutrophil granules (red circles). (a) Tumor cells trapped in these NETs would get exposed to high local concentrations of neutrophil elastase and other factors that induce cell proliferation. (b) NETs could also provide large amounts of matrix metalloproteinase-9 and serine proteases that would release vascular endothelial growth factor to promote angiogenesis. (c) NETs released on the vascular endothelium in response to inflammation could trap tumor cells allowing them to more easily arrest and extravasate the blood circulation into prometastatic sites.

As discussed earlier, many tumors presenting TANs are associated with poor prognosis. In many of these tumors, free DNA has been found. Thus the presence of NETs in these tumors most certainly would be associated with tumor progression. Supporting this idea, there are studies looking at the phenotype of TANs during tumor development. In one study, neutrophil depletion at 14 days after implantation of Lewis lung carcinoma (LLC) and AB12 mesothelioma tumors resulted in reduced tumor growth. In contrast, neutrophil depletion at 7 days after implantation had no effect on tumor growth. TANs from early tumors were more cytotoxic toward tumor cells, while TANs from established tumors acquired a more protumorigenic phenotype [64]. Moreover, in initial tumors, TANs were found in the periphery of the tumor, but in mature tumors TANs and free DNA were within the tumor [64]. In another study, sparc^−/−^ mice had defective collagen assembly within secondary lymphoid organs. This defect caused an uneven compartmentalization of lymphoid and myeloid populations that led to aberrant interactions between NETs and B cells. Under these conditions, NETs induced B cell proliferation and inhibition of apoptosis, resulting in malignant transformation [208]. Together, these data support a model for primary tumor development. Neutrophils would migrate to the new tumor and there TANs would produce NETs, which would promote tumor growth (Figure 5).

Although evidence strongly indicates that NETs within primary tumors can promote tumor progression, no mechanism for this effect has been revealed yet. However, because NETs are made of chromatin fibers decorated with antimicrobial proteins such as neutrophil elastase, cathepsin G, and MPO, it is very likely that NETs concentrate these factors to high local concentrations within the tumor microenvironment. As discussed above, these factors have all been implicated in tumor promotion. Therefore, NETs may be a way to enhance exposure of tumor cells to these bioactive proteins and in turn increase proliferation, inhibit apoptosis, and induce migration (Figure 5).

## 7. Therapeutic Approaches

Although in many instances the presence of neutrophils in tumors has a negative effect in cancer disease, these cells clearly have the capacity to destroy tumor cells. Several novel therapeutic approaches are being considered to enhance the antitumor potential of neutrophils or to block the access of TANs into growing tumors. These approaches are briefly described next.

### 7.1. Activation of Neutrophils

N1 type murine neutrophils display an activated phenotype that leads to tumor control. In consequence, tumor cells modified to express G-CSF induced recruitment of neutrophils that were able to inhibit tumor growth [157]. Activation of neutrophils with G-CSF and IFN-*β* can generate cells with an antitumor phenotype [114]. Due to the important role of neutrophils in antimicrobial responses, general activation of these cells is not good therapeutic approach since highly activated neutrophils without targeting specificity could cause excessive tissue damage.

The two types of TANs, N1 and N2, suggest that the tumor microenvironment could be manipulated to generate more antitumor TANs. This idea is supported by studies in murine cancer models where inhibition of TGF-*β* induced the appearance of antitumor neutrophils. These cells produced high levels of proinflammatory cytokines and could kill tumor cells [15].

### 7.2. Inhibition of Neutrophil Infiltration into Tumors

Another therapeutic approach aims to block infiltration of neutrophils into tumors. As indicated before, several tumors produce chemokines, mainly IL-8, which recruits neutrophils to the tumor. The use of IL-8 antagonists (such as the fully humanized neutralizing monoclonal antibody ABX-IL8) to IL-8 was shown to reduce tumor growth, metastasis, and angiogenesis of melanoma [209] and lung cancer [75]. Because other chemokines also interact with the receptors CXCR1 and CXCR2 [77], a more effective way to block neutrophil migration may be the inhibition of these receptors. Specific inhibitors for these receptors are now being developed with the idea of preventing neutrophil infiltration and retarding tumor progression [210]. For example, the CXCR2 receptor antagonist, GSK135756, is being considered to be used as an anti-inflammatory drug for chronic obstructive pulmonary disease. If GSK135756 is approved, it could have anticancer potential [211]. Another small-molecule inhibitor for CXCR1 is reparixin. This inhibitor has shown to efficiently block neutrophil recruitment into tissues and to selectively target human breast cancer stem cells in xenograft models in mice [212].

### 7.3. Inhibition of Neutrophil-Specific Enzymes

In addition to blocking neutrophil infiltration, inhibition of particular neutrophil-specific enzymes known to promote tumor progression is another therapeutic avenue being explored. For example, inhibition of NE was able to reduce significantly the growth of lung adenocarcinomas in a mouse model [91]. Also inhibition of MMP has been tried to prevent tumor angiogenesis. The bisphosphonate zoledronic acid, a strong MMP inhibitor, blocked MMP-9 expression and metalloprotease activity reducing angiogenesis and cervical cancer burden [213]. However, in other models and clinical trials, inhibition of MMP-9 was not effective at reducing tumor growth [214, 215].

### 7.4. Therapeutic Antibodies for ADCC

A more promising approach is the use of antitumor monoclonal antibodies (mAbs) to activate the ADCC potential of neutrophils. Upon Fc receptor activation, neutrophils produce ROS and release mediators with direct antitumor potential [216]. Today most mAbs used in immunotherapy belong to the IgG1 class, and they are effective at activating NK cells via Fc*γ*RIIIa (CD16a) [181]. In contrast, neutrophils activate ADCC via Fc*γ*RIIa (CD32) by preferentially engaging IgG2 class antibodies [185]. This IgG2-mediated ADCC was influenced by the functional Fc*γ*RIIa-R131H polymorphism and was induced more effectively by neutrophils from Fc*γ*RIIa-131H homozygous donors than from Fc*γ*RIIa-131R individuals [185]. Based on these findings, it has been proposed that Fc receptor polymorphisms could be biomarkers for EGFR antibodies such as Panitumumab, the only human IgG2 antibody approved for immunotherapy and inhibition of EGFR [217]. Therefore, there is a big interest in developing new improved antibodies through Fc engineering technologies in order to potentiate Fc*γ*R-mediated functions [218]. Based on this methodology, it was possible to change the ability of an Fc*γ*RIII-optimized (for NK cell) anti-EGFR antibody to efficiently activate neutrophil ADCC against EGFR-expressing tumors [219].

In addition to Fc*γ*RIIa, IFN-*γ*-activated neutrophils can perform ADCC against tumors [186]. However, it seems that Fc*α*RI (CD89) is a more potent inducer of ADCC by neutrophils [188, 189]. Thus, it has been proposed that a new generation of cancer therapeutic mAb should include IgA class antibodies to fully take advantage of the cytotoxic potential of neutrophils [220]. Indeed, this idea is supported by a new IgA2 anti-EGFR antibody derived from the IgG anti-EGFR mAb cetuximab. IgA2 EGFR was more effective than cetuximab* in vivo* against EGFR-transfected Ba/F3 target cells [221]. Very recently, it was also shown that the combination of IgG and IgA mAbs to two different tumor targets (EGFR and HER2) led to enhanced cytotoxicity compared with each isotype alone [222].

## 8. Conclusion

Tumor development is influenced by many different host cell types. It has become clear that many tumors present infiltrating neutrophils. The exact role for these tumor-associated neutrophils (TANs) has yet to be completely elucidated. Early reports showed that neutrophils could be cytotoxic to tumor cells. However, a tremendous body of clinical evidence has shown that neutrophils promote tumor progression in various ways. Neutrophils can induce tumor proliferation and angiogenesis and can enhance tumor cell migration and metastasis. Yet, a type of TANs, named N1, can indeed display antitumor functions. New therapeutic ways to recruit and activate these N1 type neutrophils are being investigated in order to turn protumorigenic neutrophils into antitumor effector cells. Blocking neutrophil-derived components known to help tumor growth is a field of active research. Also, very promising results have been found with the use of therapeutic antibodies, which induce neutrophils to perform ADCC and to release cytokines that modulate the immune response against tumors. New antibodies are being designed so that they have better affinity for particular Fc receptors and induce stronger antitumor responses. Learning how to flip the neutrophil coin to “the winning side,” namely, functioning as antitumor effector cells, is a challenge for future research that will certainly provide us with new therapeutic options for cancer treatment.
